# Temporomandibular joint disc displacement with reduction: a review of mechanisms and clinical presentation

**DOI:** 10.1590/1678-7757-2018-0433

**Published:** 2019-02-21

**Authors:** Rodrigo Lorenzi POLUHA, Giancarlo De la Torre CANALES, Yuri Martins COSTA, Eduardo GROSSMANN, Leonardo Rigoldi BONJARDIM, Paulo César Rodrigues CONTI

**Affiliations:** 1 Universidade de São Paulo, Faculdade de Odontologia de Bauru, Departamento de Prótese, Grupo de Dor Orofacial de Bauru, Bauru, São Paulo, Brasil.; 2 Universidade de Campinas, Faculdade de Odontologia de Piracicaba, Departamento de Ciências Fisiológicas, Piracicaba, São Paulo, Brasil.; 3 Universidade Federal do Rio Grande do Sul, Faculdade de Odontologia, Departamento de Ciências Morfológicas, Porto Alegre, Rio Grande do Sul, Brasil.; 4 Universidade de São Paulo, Faculdade de Odontologia de Bauru, Departamento de Ciências Biológicas, Seção de Fisiologia da Cabeça e da Face, Bauru, São Paulo, Brasil.

**Keywords:** Temporomandibular joint, Temporomandibular joint disc, Temporomandibular joint disorders

## Abstract

Disc displacement with reduction (DDWR) is one of the most common intra-articular disorders of the temporomandibular joint (TMJ). Factors related to the etiology, progression and treatment of such condition is still a subject of discussion. This literature review aimed to address etiology, development, related factors, diagnosis, natural course, and treatment of DDWR. A non-systematic search was conducted within PubMed, Scopus, SciELO, Medline, LILACS and Science Direct using the Medical Subjective Headings (MeSH) terms “temporomandibular disorders”, “temporomandibular joint”, “disc displacement” and “disc displacement with reduction”. No time restriction was applied. Literature reviews, systematic reviews, meta-analysis and clinical trials were included. DDWR is usually asymptomatic and requires no treatment, since the TMJ structures adapt very well and painlessly to different disc positions. Yet, long-term studies have shown the favorable progression of this condition, with no pain and/or jaw locking occurring in most of the patients.

## Introduction

According to the American Academy of Orofacial Pain, temporomandibular disorder (TMD) is defined as a group of disorders involving the masticatory muscles, the temporomandibular joint (TMJ), and the associated structures.^1^ The most common TMJ conditions are pain-related and intra-articular disorders.^2^ Intra-articular disorders of the TMJ have been defined as an abnormal positional relationship between the disc and the condyle, articular eminence, and/or articular fossa.^3^

**Figure 1:**
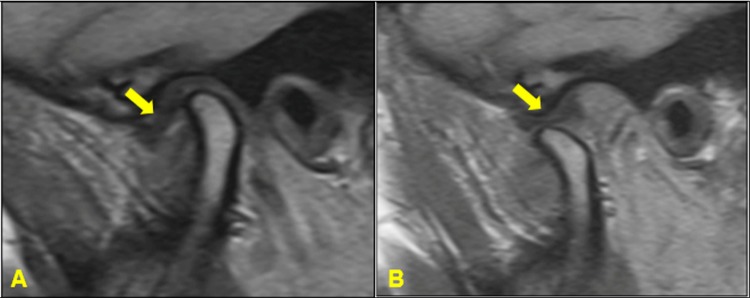
Disc displacement with reduction (DDWR). A: Closed mouth, the articular disc (yellow arrow) is anteriorly displaced in relation to the condyle; B: Open mouth, the disc (yellow arrow) returns to the intermediate area

Among the intra-articular disorders of the TMJ, disc displacement with reduction (DDWR) corresponds to 41% of TMD clinical diagnoses.^4^ Also, DDWR can occur in 33% of asymptomatic individuals.^5^ In patients with DDWR, when the mouth is closed, the articular disc is displaced in relation to the condyle and, when the mouth is open, the disc returns to the intermediate area between the condyle and the articular tubercle^1^ ^,^ ^6^ (Figure 1).

Although the disc can be displaced in any direction (i.e., anterior, posterior, lateral, or medial),^7^ posterior and pure sideways displacements seem to be rare, whereas anterior displacement appears to be the most common.^8^ After the disc reduces during condylar translation, the range of motion is not limited; however, mandibular movements may not be as smooth as in a regular condition because of the momentary sliding of the condyle on and off of the disc.^9^ Notwithstanding, once the mouth opening position is achieved, the final position of the condyle and the disc of a joint with DDWR is almost identical to one without displacement.^10^

**Figure 2:**
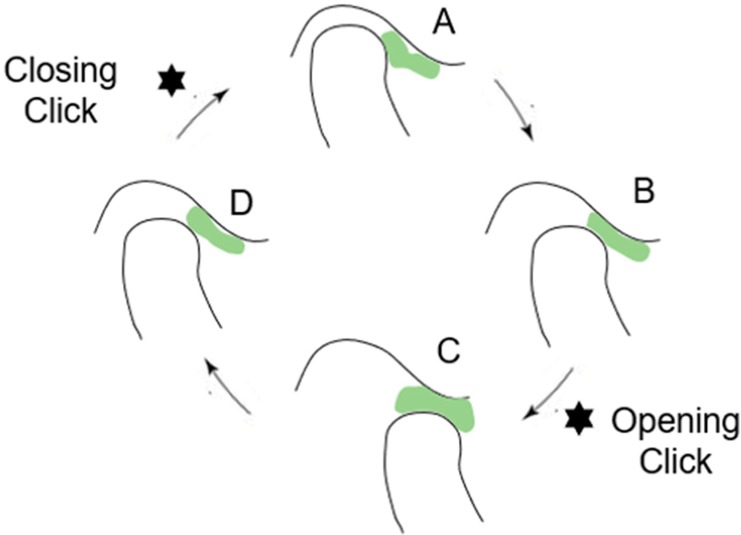
Disc displacement with reduction (DDWR). A: Articular disc anteriorly displaced with retrodiscal fibrosis (red arrow); B: Reduced disc, retrodiscal fibrosis (red arrow)

Clinically, DDWR is related to TMJ noise.^11^ The movement of the disc onto and off may result in a clicking, snapping, and/or popping sound known as opening and closing click^12^ (Figure 2). TMJ clicking corresponds to 26.2% of clinical signs of TMD and is one of the most common complaints of patients.^13^ Self-report of TMJ clicking are more frequent in care-seeking patients that also have greater non-specific physical symptoms, with a propensity to somatization and with the heightened awareness of their own body image.^14^

The correlation between joint pain, known as arthralgia, and disc position is still a matter of debate.^15^ Although most cases of DDWR are not accompanied by pain, some sort of joint inflammation may occur and cause painful symptoms;^9^ even those asymptomatic DDWR joints can present some sensitization level. A study evaluating the pressure pain threshold (PPT), showed that individuals with asymptomatic DDWR had significant lower PPT values than individuals with a proper disc/condyle relationship (1.64±0.40 kgf/cm^2^ and 2.35±0.56 kgf/cm^2^, respectively), which indicates that, despite the adaptive tissue capacity of the individual with DDWR, there is a certain degree of modification in the response to the pressure application.^16^ This fact, however, is not *per se* indicative of the need for therapy.

DDWR is a highly prevalent clinical condition^4^ that still raises many doubts to patients and clinicians regarding the true risk involved in the disorder, odds of progression and need for treatment. Therefore, this literature review aimed at addressing the recent advances in the etiology, epidemiology, diagnosis, natural course, and management of DDWR.

## Methodology

A non-systematic search was conducted to identify articles related to the etiology, epidemiology, diagnosis, and treatment of DDWR. To this goal, searches within the PubMed, Scopus, SciELO, Medline, LILACS and Science Direct were performed using the Medical Subjective Headings (MeSH) terms “temporomandibular disorders”, “temporomandibular joint”, “disc displacement”, and “disc displacement with reduction”. Literature reviews, systematic reviews, meta-analysis, observational studies and clinical trials published between March 1987 and June 2018 were included.

## Literature review

### Etiology of DDWR

Etiological factors leading to DDWR are partially attributed to abnormal biomechanical forces applied to the mandibular condyle, which alter the shape and function of the articular tissues.^17^ The literature cites many potential factors that increase the risk of DDWR such as chronic (microtrauma) or acute injuries (macrotrauma) directed against the TMJ, shape and/or dynamic properties alterations of the TMJ components, lack of lubrication, form of the disc modifications, degenerative articular disorder, some occlusal abnormalities, hyperactivity of the lateral pterygoid muscle, joint hypermobility, weakness or laxity of the TMJ ligament and joint capsule.^7^ ^,^ ^17^ ^-^ ^19^

The disc-condyle complex derangement comes from the alteration of the relation of the disc on the mandibular condyle.^7^ This loss of normal disc movement can occur when there is elongation of the discal collateral ligaments and the inferior retrodiscal lamina.^20^ Also, a thinning of the posterior border of the disc may allow the disc to be displaced in a more anterior position.^20^ With the condyle resting on a more posterior portion of the disc or retrodiscal tissues, an abnormal translatory shift of the condyle over the posterior border of the disc can occur during opening.^20^ Because the opening movement relocates the disc in the joint, this stage is named DDWR.^1^ ^,^ ^7^

In the past, it was believed that the disc could be positioned more anteriorly by traction of the lateral pterygoid muscle (LPM).^20^ This theory, however, was disproved based on the assumption that only a small amount of muscle fibers of the LPM are able to transpose the joint capsule and attach directly into the disc.^21^ This weak connection also weakened the theory of LPM muscle contraction involvement in the genesis of disc displacement^21^. In a study with magnetic resonance imaging (MRI), evaluating the correlation between the LPM muscle attachment type and internal derangement of the TMJ, no statistically significant correlation was found.^22^

The prevalence of DDWR is higher in female patients.^23^ This fact may derive from the influence of some female-specific characteristics such as greater joint laxity,^24^ and greater intra-articular pressure.^25^ There is an association between age and DDWR.^26^ A multiple logistic regression analysis for DDWR showed that this condition was related to an increased age.^27^ Since joint morphology and intra-articular spatial dimensions change with increasing age, the space insufficiency within the TMJ may develop, leading to a disc positional change.^27^ ^,^ ^28^

TMJ hypermobility, defined as condylar translation beyond the eminence at maximum mouth opening, has also been positively correlated with DDWR.^29^ Occlusal related factors were considered important factors for TMJ internal derangements for a long time. This association, however, has been proved not to be as consistent as thought in the past. By evaluating occlusal factors in children and teenagers (10.8*±*3.9 years), a study showed that an increasing overbite [odds ratio (OR)=1.15] could be considered a risk factor for DDWR.^30^ However, in an adult population (32.2*±*5.7 years), the contribution of occlusal features to DDWR was considered minimal, with no clinical relevance.^31^

The influence of oral habits on the disc position has also been addressed. An experimental study with DDWR patients submitted to an intensive chewing exercise showed that this activity may strongly delay or even hamper the disc reduction on mouth opening.^32^ Nocturnal tooth grinding has not found to be a risk factor for DDWR.^32^ However, several patients’ reports with disc displacement (without distinguishing between those with reduction or without reduction) suggest that clenching and grinding, especially at daytime, are positively correlated with disc displacement.^5^ ^,^ ^33^ ^,^ ^34^ Nevertheless, these finds should be viewed with caution, because none of them used a gold standard tool such as polysomnography or portable diagnostic devices for measuring electromyography activity of masticatory muscles.

TMJ anatomy is also correlated with disc displacement development. Condylar volumes (cortical and trabecular components) are significantly associated with disc displacement and all condylar volumes decrease as disc displacement progress from reduction to non-reduction in both genders.^35^ An association was also observed between the shape of the articular eminence (sigmoid form presented the greatest incidence), disc configuration (biconcave shape was the most common), disc position (anterosuperior position was the most frequent) and DDWR.^36^ ^,^ ^37^

### Natural course of DDWR

**Figure 3:**
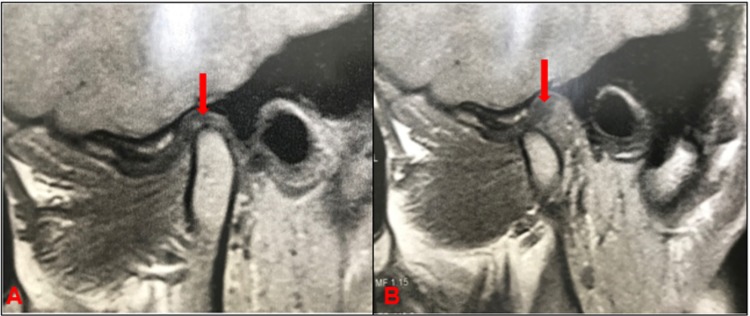
Disc displacement with reduction (DDWR). A: Closed mouth, the articular disc is displaced; B: Mouth opening, followed by an opening click; C: Open mouth, the articular disc is reduced. D: Mouth closing, followed by a closing click

It has been suggested that DDWR would be the first stage of disc displacement, possibly evolving to disc displacement without reduction (DDWoR).^38^ Nevertheless, such observation is not consistent to all conditions and types of DDWR that really evolve to DDWoR.^17^ ^,^ ^39^ DDWR is considered stable if there are no complaints regarding intermittent locking in patient’s history.^40^ Actually, DDWR can remain stable for years depending on adaptive physiological processes that may occur.^39^ The main disc adaptive physiological process is the retrodiscal fibrosis. In a study evaluating 80 TMJs in 40 pain-free individuals (mean age 28.5 years), all TMJs analyzed the retrodiscal fibrosis, being significantly more evident in the 44 TMJs with DDWR.^41^ Fibrosis involves the bilaminar zone of the TMJ as well as the interposed vascular and fatty tissue; it presents a markedly low signal intensity, a homogeneous structure in proximity to the disc, it may not be distinguished from the disc, and has an inhomogeneous aspect, whereas gradually deviating posteriorly from the disc.^42^ It is possible that this retrodiscal fibrosis explains why most DDWR are painless (Figure 3).

A study with 81 TMJ from patients with DDWR (mean age 19.65 years) showed no diagnosis change after a follow-up of 9.47 months.^43^ Another study with 92 TMJ from patients with DDWR (mean age 20.76 years) that underwent a follow-up of 9.38 months without treatment, showed that only 6 (6.5%) TMJ evolved into DDWoR. In the remainder, 86 (93.5%) DDWR temporomandibular joints, the disc length was evaluated, and the results showed a reduction of 0.66±0.97 mm after the follow-up (in the same study, 131 TMJ with DDWoR showed a decrease of 1.36±1.48 mm in disc length).^44^ A recent longitudinal study showed that of the 155 TMJ that had baseline diagnoses of normal/indeterminate or DDWR (and who received no treatment), 137 (88.4%) had no change in diagnosis 7.9 years later, on average; this result collaborates with previous studies that state that the classic model of DDWR progression to another internal derangement (such as DDWoR) is uncommon.^45^

A study with 24 patients with DDWR who did not undergo treatment showed, after 25.8 months of follow-up, that the range of mandibular movement remains unchanged over time and the clicking does not progress to locking in most patients.^39^ Another study, also with a two-year follow-up, showed that the disappearance of clicking associated with DDWR can be related to a gradual loss of the TMJ disc reduction capacity, and that the reports on intermittent locking in patients with DDWR are indicative of the development of DDWoR. However, this loss is only rarely accompanied by symptoms of permanent locking.^40^

Lundh and collegues^46^ (1987), evaluated 70 patients with reciprocal TMJ clicking, during a three-year follow-up without treatment. Reciprocal clicking remained unchanged in 50 (71%) and disappeared in 20 patients (29%). Fourteen patients (20%), in whom clicking disappeared, attained normal mouth opening, whereas the locking was developed in 6 patients (9%). Another study evaluated the changes in TMJ clicking in 190 subjects with clicking or popping of the TMJ and myofascial pain dysfunction. The patients were originally treated by conservative, nonsurgical modalities that were not specifically directed to the problems of TMJ noise or disc derangement. The follow-up period ranged from 1 to 15 years. In terms of clicking status, 126 patients (63%) reported cessation or improvement, and 74 patients (36%) were unchanged. Only three patients described this symptom as worse.^47^

### Diagnosis of DDWR

Many studies have evaluated the diagnostic methods of DDWR.^2^ ^,^ ^48^ ^,^ ^49^ MRI is considered the gold standard exam for TMJ conditions, since it allows the simultaneous evaluation of the morphology and position of the articular disc and bone structures of the TMJ, in addition to evaluating the functional relationships between the condyle, articular disc, mandibular fossa, and articular eminence.^50^ A multi-section analysis of MRI images allows distinguishing the correct disc position from disc displacement and can improve the ability to distinguish between various stages of intra-articular derangement of TMJ.^51^ For DDWR diagnosis, MRI showed a specificity range between 88 and 90% and sensitivity range between 78 and 83.3%.^48^ ^,^ ^52^

Over the last decade, ultrasonography (US) of the TMJ has also been the focus of an increasing number of studies, which aimed to assess the diagnostic accuracy of US for TMJ disc displacement.^53^ Compared to MRI, US showed a sensitivity of 78.6%, specificity of 66.7%, and accuracy of 73.0% for DDWR diagnosis.^54^ US is a valuable imaging technique in assessing TMJ disc position, however, the diagnostic value of high-resolution US depends strictly on the examiner’s skills and on the equipment used.^55^ Hence, diagnosis with US still requires standardization of the method as well as further research to confirm its effectiveness.^49^

Manual TMJ inspection is one of the most common techniques to assess TMJ dysfunctions.^1^ ^,^ ^56^ Because DDWR only becomes clinically relevant when it interferes with TMJ function, the clinical diagnostic approach can be considered a benchmark for DDWR recognition.^12^ The Diagnostic Criteria for Temporomandibular Disorder (DC/TMD),^2^ which is a standardized tool to diagnosis TMD, presents the following diagnosis criteria for DDWR: Positive for at least one of the following: − In the last 30 days, any TMJ noise(s) present with jaw movement or function; OR − Patient report of any noise present during the exam. Positive for at least one of the following: − Clicking, popping, and/or snapping noise during both opening and closing movements, detected with palpation during at least one of three repetitions of jaw opening and closing movements; OR − Clicking, popping, and/or snapping noise detected with palpation during at least one of three repetitions of opening or closing movement(s); AND − Clicking, popping, and/or snapping noise detected with palpation during at least one of three repetitions of right or left lateral, or protrusive movement(s). In a recent meta-analysis, the validity of clinical protocols compared with MRI performed in studies evaluating only DDWR presented sensitivity of 44% (39 to 49%) and specificity of 51% (46 to 57%). The value of the area under the curve for validity of clinical protocols was 0.56 for studies evaluating DDWR.^57^

### Treatment of DDWR

Clinically, DDWR can be considered in three ways: (I) as a clinical finding, when the patient has no complaints and the clicking is identified during the manual professional TMJ inspection or the disc displacement is diagnosed by an MRI examination and only orientations to the patient are sufficient; (II) as the main patient’s complaint, when the noise motivates the patient to seek treatment and the treatment plan should address options to reduce/eliminate the clicking; (III) when the click is accompanied by arthralgia and the treatment plan should be focused on the amelioration of pain.

Treatment of DDWR, especially when the noise is the complaint, is not an easy task, and for the vast majority of individuals, an explanation of the situation, along with avoidance of overloading activities is the best option. As mentioned above, there is evidence for a self-limiting tendency to this condition, without any kind of clinical conduct or treatment.^17^ ^,^ ^58^ In absence of complaints, no treatment is recommended.^39^ It is important to remember that there is still no gold standard treatment for TMJ clicking; thus, when patients complain about the noises (clicking) and seek treatment, conservative approaches are always the first choice.^17^

Amongst conservative treatments there are: patient education (such as the explanation about DDWR and orientation of avoidance of excessive open mouth), exercises, relaxation techniques, and occlusal splints.^17^ ^,^ ^59^ Au and Klineberg^60^ (1993), used isokinetic exercises of the jaw in 22 patients complaining about TMJ clicking. The exercises included two movement sequences as follows: (1) jaw opening and closing over a distance of 15 mm, and (2) moving the jaw to the left and right over a distance of 5 mm to each side. Jaw movements were performed against a constant but moderated resistance provided by the subject’s hand. After six months of follow-up, the clicking disappeared in approximately 82% of the patients. Yoda, et al.^61^ (2003), also find a clicking reduction on 61.9% TMJ of patients with painless clicking, after three months of therapeutic exercise (protrusion and retrusion). Huang, et al.^62^ (2011), treated 59 patients with painless clicking with a mandibular stabilization occlusal splint (hard acrylic). After six months, there was an elimination of TMJ clicking in 71.2% of cases. These findings were like those previously found by Conti, et al.^63^ (2006), who treated with occlusal devices 57 patients with a complaint about TMJ pain and clicking. The patients were divided into three groups: bilateral balanced occlusal splints; canine guidance occlusal splints; and, nonoccluding splint. All subjects had a general improvement on the pain, though subjects in the occlusal splint groups had better results than subjects in the nonoccluding splint group. The frequency of joint noises decreased over time, with no significant differences among groups. In another study, Conti, et al.^64^ (2015) assessed the effectiveness of the partial use of intraoral devices and counseling in the management of 60 patients with DDWR and arthralgia. The patients were equally divided into three groups: group I wore anterior repositioning occlusal splints; group II wore NTI-tss devices, and group III only received counseling for behavioral changes and self-care. The first two groups also received the same counseling. At the beginning of the study, all patients had a TMJ click at least on one side. When joint sounds (clicking) were investigated after 3 months, a decrease in frequency for groups I and III were observed. On the other hand, an increase in frequency for those in group II was detected, although with no significance.

Even though, there is no direct scientific evidence that impaired lubrication of TMJ is indeed responsible for the development of a disc displacement.^65^ Hyaluronic acid (HA), has been suggested as an alternative therapeutic agent for the management of internal TMJ derangements.^66^ Basterzi, et al.^67^ (2009), treated 20 patients with DDWR, with clicking complaint, with intraarticular hyaluronic acid (HA) injections (at weekly intervals for 3 weeks). After one year, there was a significant reduction of joint noises, however, there was no control group without intervention. Korkmaz, et al.^68^ (2016), compared the effectiveness of a single HA injection, a double HA injection, and splint therapy for the treatment of DDWR. The results of this study showed that HA injection and stabilization splinting are acceptably successful modalities of treatment to alleviate the clinical signs and symptoms of DDWR (double HA injection seems to be superior), especially in reducing TMJ clinking.

Invasive methods should be considered only after failed attempts at conservative care in what concerns persistence of complaints,^9^ and are rarely indicated for DDWR, when noises are considered. These modalities comprise TMJ arthrocentesis, arthroscopies and surgical techniques.^17^ Surgical procedures always involve some risks such as extravasation of liquid to the surrounding tissue, lesion of the facial nerve, optical lesion, pre-auricular hematoma, arteriovenous fistula, trans articular perforation, intracranial perforation, extradural hematoma and post-surgical intra-articular problems, which justify the clinician’s caution to recommend any of such procedures as routine or first treatment choice.^69^ ^,^ ^70^

## Conclusion

DDWR is the most common of the TMJ disc displacements. It is commonly an asymptomatic condition and no treatment is usually required, since the structures in this region may adapt and the progression is extremely benign for most cases. Treatment should be done when DDWR is the patient’s main complaint, and when the noise motivates the patient to seek treatment and/or the click is accompanied by pain.
