# Erratum

**DOI:** 10.1590/1678-7757-2019er001

**Published:** 2019-03-27

Due to a publishing error the article: “Temporomandibular joint disc displacement with reduction: a review of mechanisms and clinical presentation”, published at Journal of Applied Oral Science 2019;27:e-20180433 was issued with the following error:

**Figure 2:**
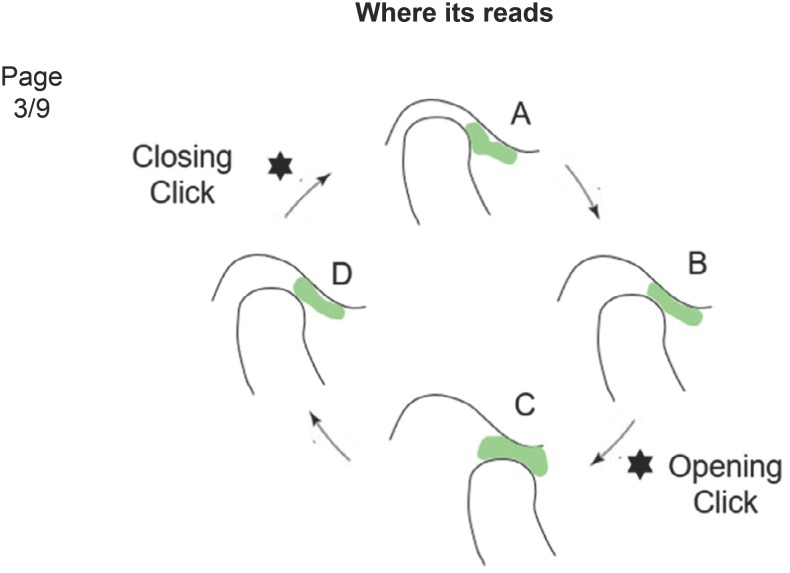
Disc displacement with reduction (DDWR). A: Articular disc anteriorly displaced with retrodiscal fibrosis (red arrow); B: Reduced disc, retrodiscal fibrosis (red arrow)

**Figure 2:**
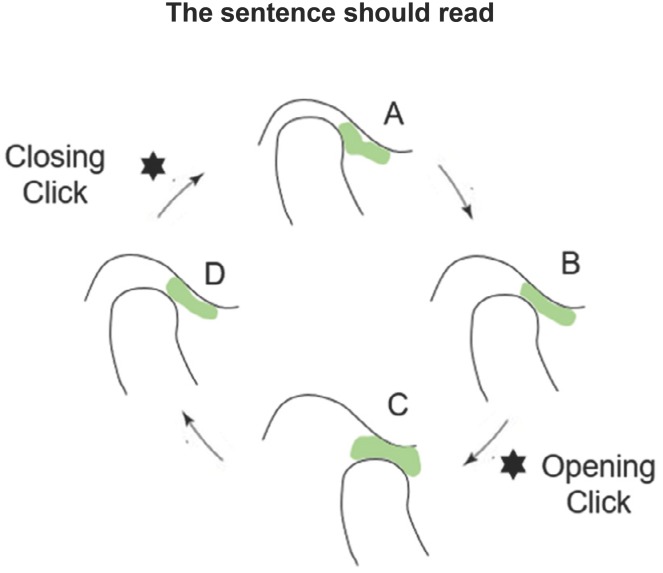
Disc displacement with reduction (DDWR). A: Closed mouth, the articular disc is displaced; B: Mouth opening, followed by an opening click; C: Open mouth, the articular disc is reduced. D: Mouth closing, followed by a closing click

**Figure 3:**
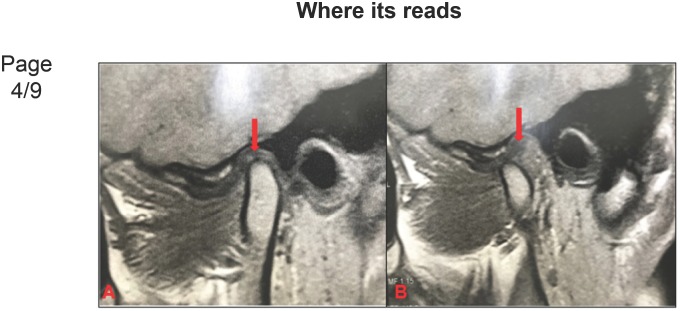
Disc displacement with reduction (DDWR). A: Closed mouth, the articular disc is displaced; B: Mouth opening, followed by an opening click; C: Open mouth, the articular disc is reduced. D: Mouth closing, followed by a closing click

**Figure 3:**
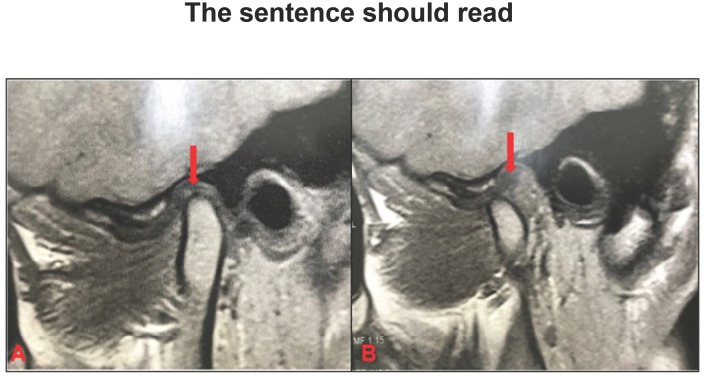
Disc displacement with reduction (DDWR). A: Articular disc anteriorly displaced with retrodiscal fibrosis (red arrow); B: Reduced disc, retrodiscal fibrosis (red arrow)

